# Prevalence and Characteristics of Interventional Trials Conducted Exclusively in Elderly Persons: A Cross-Sectional Analysis of Registered Clinical Trials

**DOI:** 10.1371/journal.pone.0155948

**Published:** 2016-05-19

**Authors:** Florence T. Bourgeois, Karen L. Olson, Tony Tse, John P. A. Ioannidis, Kenneth D. Mandl

**Affiliations:** 1 Department of Pediatrics, Harvard Medical School, Boston, Massachusetts, United States of America; 2 Department of Emergency Medicine, Harvard Medical School, Boston, Massachusetts, United States of America; 3 Computational Health Informatics Program, Boston Children’s Hospital, Boston, Massachusetts, United States of America; 4 National Center for Biotechnology Information, National Library of Medicine, Bethesda, Maryland, United States of America; 5 Stanford Prevention Research Center, Department of Medicine, Stanford University School of Medicine, Stanford, California, United States of America; 6 Department of Health Research and Policy, Stanford University School of Medicine, Stanford, California, United States of America; Mario Negri Institute for Pharmacology Research, ITALY

## Abstract

**Background:**

Elderly patients represent the greatest consumers of healthcare per capita but have historically been underrepresented in clinical trials. It is unknown how many trials are designed to focus exclusively on elderly patients.

**Objective:**

To define the prevalence of interventional trials that study exclusively elderly persons and describe the characteristics of these trials, including their distribution across conditions most prevalent in the elderly.

**Design:**

All interventional clinical trials enrolling exclusively elderly patients (≥65 years), conducted primarily in high-income countries, and initiated between 2006 and 2014, identified through ClincialTrials.gov.

**Main Measures:**

Trials were identified and characterized according to design features and disease categories studied. Across disease categories we examined the burden of disease in the elderly in high-income countries (measured in disability-adjusted life years [DALYs]) and compared to the number of trials conducted exclusively in the elderly.

**Results:**

Among 80,965 interventional trials, 1,112 (1.4%) focused on elderly patients. Diverse types of interventions were studied in these trials (medications 33%, behavioral interventions 18%, and dietary supplements 10%) and the majority was funded by non-profit organizations (81%). Studies tended to be small (median sample size 122 participants [IQR 58, 305]), single-center studies (67%). Only 43% of 126 disease categories affecting elderly persons were studied in trials focused on the elderly. Among these disease categories, there was a 5162-fold range in the ratio of DALYs per trial. Across 5 conditions where over 80% of DALYs are in the elderly, there were a total of only 117 trials done exclusively in the elderly.

**Conclusions:**

Very few and mostly small studies are conducted exclusively in elderly persons, even for conditions that affect almost exclusively the elderly.

## Introduction

Over 40 million individuals in the United States are 65 years or older with projected increases to 56 million by 2020.[1] Approximately a quarter of the disease burden in high-income countries is borne by elderly persons; this population consumes the highest amount of healthcare per capita, with more than $530 billion spent on medical care and $56 billion on prescription drugs annually.[2,3] Trial results obtained in younger patient groups are often extrapolated to elderly patients, but the effectiveness and/or safety of interventions may differ in older individuals due to age-related pathophysiology, risk factors, disease severity and commonly used concomitant treatments.[4]

There are strong indications that the study of the elderly in clinical trials is insufficient. Many trials have explicit upper age limitations preventing enrollment of older persons while others exclude the elderly through indirect criteria such as comorbidity, cognitive impairment, or concomitant drug therapy.[4–8] As a result, a paucity of clinical trial evidence for elderly persons has been demonstrated across a number of diseases that are prevalent in the elderly, including heart failure, cancer, osteoarthritis, and diabetes.[4,6,8–11] The under-representation of the elderly in clinical research has been recognized by drug regulatory agencies, which have issued specific recommendations to increase the study of therapeutics in older patients, especially for drugs intended to treat this population.[12,13]

One approach to addressing the need for clinical evidence on elderly patients is to conduct trials that are exclusively focused on elderly patients. These trials might be designed to study specific conditions that are known to represent a large burden in the elderly. It is currently unknown what proportion of trials focuses exclusively on the elderly and how well these trials align with the diseases posing the greatest burden in older patients. Accordingly, we sought to define the prevalence of interventional trials that study exclusively elderly persons and to describe their characteristics, including their distribution across disease conditions common in the elderly.

## Methods

### Clinical trials performed exclusively in the elderly

Clinical trials performed exclusively in elderly persons were collected from the interventional trials registered in the ClinicalTrials.gov registry. Based in the United States, this is a publicly accessible, web-based registry that is maintained by the National Library of Congress on behalf of the National Institutes of Health. It represents the most comprehensive trial registry with more than 190,000 trials from 189 countries.[14] Prospective trial registration has become standard practice due to a number of policies and federal legislations that mandate registration of trials.[15–17] As a result, the registry is well-suited to the evaluation of clinical research activity and has been used to assess various aspects of trial design, selective reporting and publication of trials, globalization of the research enterprise, and the scope of study for specific drugs and interventions. [18–23]

We selected all interventional trials with a start date on or after January 1^st^, 2006 with participant age eligibility criteria indicating that the trial included exclusively elderly persons, defined as patients ≥65 years of age. We further limited our sample to those conducted primarily in high-income countries because disease burden and the age distributions of patients varies substantially between countries in different income brackets and the proportion of elderly patients is particularly high in high-income countries. All data were downloaded from ClinicalTrials.gov on February 12^th^, 2014.

Each trial was manually reviewed and mapped to one of the conditions in the WHO Global Burden of Disease study taxonomy.[24] This classification system includes 155 disease categories. Diseases that do not affect elderly persons in high-income countries were excluded, including several maternal conditions, sepsis and other infectious disorders of the newborn baby, other neonatal disorders, childhood behavioral disorders, sudden infant death syndrome, leprosy, and neglected tropical diseases, leaving 126 diseases for analysis. We excluded trials studying diseases not represented in the WHO classification system (e.g. amyloidosis) or those studying diseases that could not be mapped to a single disease entity (e.g. chronic pain).

### Burden of Disease in the Elderly

Burden of disease data for elderly persons were obtained from the Global Burden of Disease 2010 Study.[25] This study represents a comprehensive, global collaborative project that quantifies the mortality and disability related to diseases, injuries, and risk factors. Data are stratified by age, gender, and country and include a composite measure, disability adjusted life years (DALYs). This metric accounts for both years of life lived in states of disability and years of life lost due to premature death as a result of disease or injury, with one DALY representing one year of healthy life lost. Since the distribution of research activity should reflect both morbidity and mortality, this measure is particularly well suited for the assessment of the alignment between research and disease burden.[26,27]

The Global Burden of Disease 2010 Study provides DALYs for each of the diseases defined in the study. We obtained DALYs for persons ≥65 years of age residing in high-income nations. Furthermore, we identified the disease categories for which ≥80% of the burden of disease in high-income countries is accounted for by persons ≥65 of age as these diseases are most likely to require evidence from trials that focus on elderly patients.

### Trial Data Extraction and Classifications

For every trial in our sample, we collected information on the type of intervention under study (e.g. drug, device, biologic, procedure), the outcomes examined (e.g. safety, efficacy, or both), study phase, start year, whether the study was randomized and, if so, the unit of randomization (participant or cluster), the number of participants enrolled (or the estimated number if the study was ongoing), and the length of the study (calculated as the time from study start to primary completion date). The primary funding source for trials is categorized in the registry as industry, government, or other, which includes academic institutions and other non-profit research networks and organizations. We classified each trial as industry or non-industry sponsored.

We identified all trial study site countries and the total number of sites in each trial in order to determine whether a trial was conducted in a high-income country. Countries were assigned to income levels using the World Bank income categorization, which is based on a country’s gross national income per capita.[28] High-income trials were defined as those in which at least half of the study sites were located in a high-income country.

### Data Analysis

Descriptive analyses were used to characterize the trials according to different design features. Research activity in trials conducted exclusively in the elderly was measured in terms of the number of interventional trials as well as the number of trials that were randomized, that were randomized and studied a drug intervention, and that were funded primarily by industry. We also considered the number of participants enrolled in trials, limiting to those that were randomized at the participant level as opposed to cluster-randomized. For each disease category studied in at least one trial, we calculated the ratio of DALYs per trial in order to compare the allocation of research activity in trials focused exclusively on the elderly across disease categories. The correspondence between research activity and burden of disease was assessed using Spearman’s rank-order correlation. All statistical analyses were performed using SAS software, version 9.3 (SAS institute, Inc., Cary, NC).

## Results

**Fig 1:**
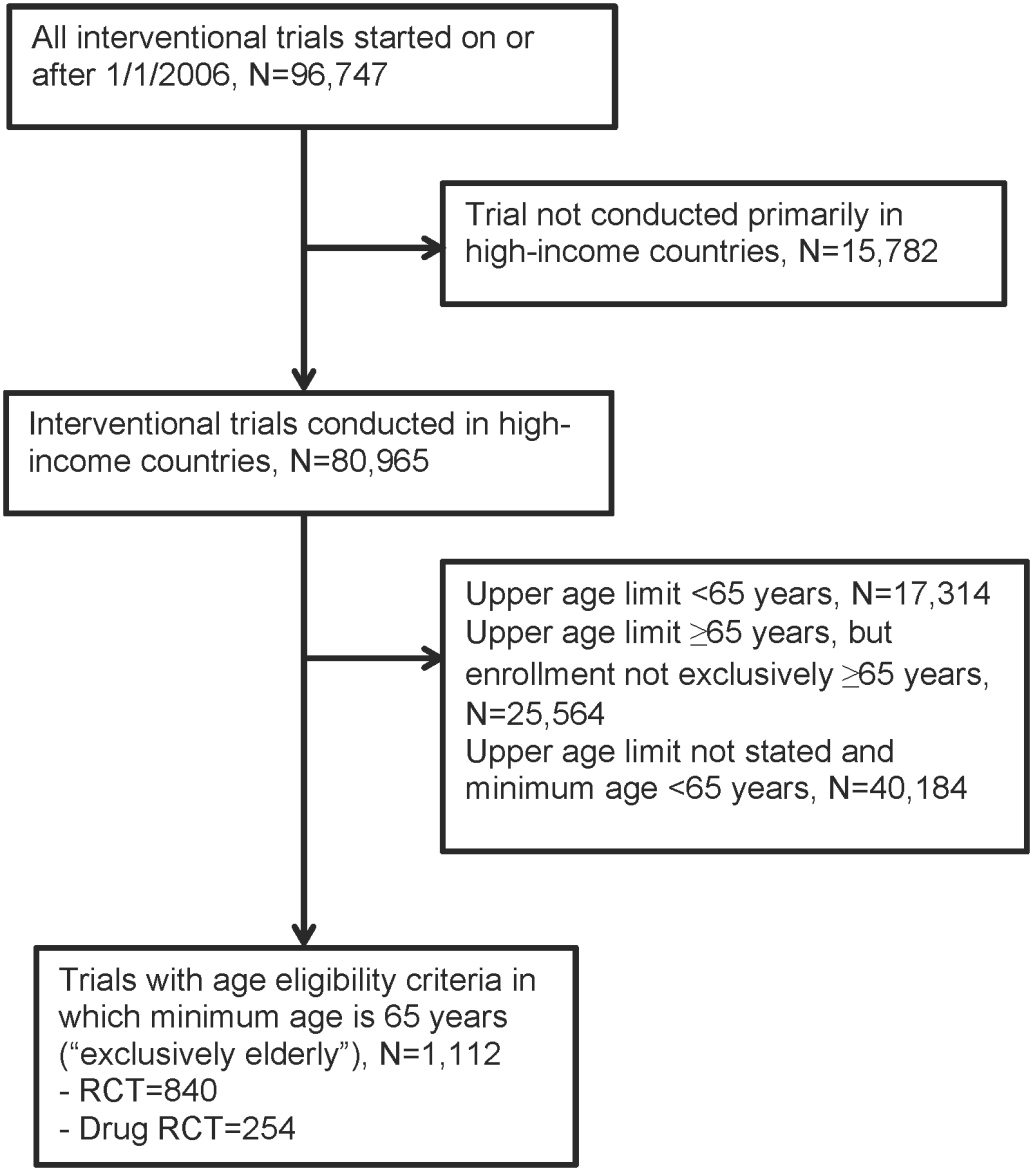
Flowchart for inclusion of clinical trials registered in ClinicalTrials.gov and focusing on elderly persons.

Among 80,965 interventional trials conducted in high-income countries, we identified 1,112 (1.4%) trials with an eligibility age range that was exclusively elderly (Fig 1). Among these trials, there were 840 RCTs.

Tables 1 and 2 describe the characteristics of the trials. The most common intervention type was drugs (33%) followed by behavioral interventions (18%), dietary supplements (10%), devices (8%), and biologics (7%). Most (58%) of the trials addressed treatment and 81% were funded primarily by non-industry sources. The study design was randomized in three-quarters of trials with 46% designed as open label trials and another 22% as single-blind trials. Two-thirds were conducted at single centers and the median number of trial locations was 1 (IQR 1, 2). The median trial sample size was 122 (IQR 58, 305) participants and the median study length 24 months (IQR 12, 38).

**Table 1:** General characteristics of registered interventional trials studying exclusively elderly persons.

Characteristic	Trials studying exclusively elderly persons, N = 1,112
**Intervention type, N (%)**	
Drug	368 (33.1)
Device	87 (7.8)
Behavioral	195 (17.5)
Procedure	70 (6.3)
Biologic	78 (7.0)
Dietary supplement	108 (9.7)
Radiation	7 (0.6)
Genetic	1 (0.1)
Other	198 (17.8)
**Primary Purpose, N (%)**	
Treatment	579 (52.1)
Prevention	264 (41.1)
Supportive care	64 (5.8)
Health services research	57 (5.1)
Diagnostic/screening	42 (3.8)
Basic science	29 (2.6)
Not stated	75 (6.8)
**Primary Funding Source, N (%)**	
Industry	210 (18.9)
Non-industry	902 (81.1)
**Start Year, N (%)**	
2006–2007	204 (18.5)
2008–2009	248 (22.3)
2010–2011	307 (27.6)
2012–2014	353 (31.7)

**Table 2:** Study design characteristics of registered interventional trials studying exclusively elderly persons.

Characteristic	Trials studying exclusively elderly persons, N = 1,112
**Study Phase**^1^, **N (%)**	
Phase 1	92 (8.3)
Phase 2	222 (20.0)
Phase 3	156 (14.0)
Phase 4	127 (11.4)
Not stated	515 (46.3)
**Interventional Model, N (%)**	
Single group	244 (24.4)
Parallel	728 (65.5)
Crossover	59 (5.3)
Factorial	36 (3.2)
Not stated	18 (1.6)
**Allocation, N (%)**	
Randomized	840 (75.5)
Non-randomized	272 (24.5)
**Masking**	
Open label	516 (46.4)
Single-blind	244 (21.9)
Double-blind	336 (30.2)
Not stated	16 (1.4)
**Study outcomes, N (%)**	
Efficacy and safety	330 (29.7)
Efficacy only	454 (40.8)
Safety only	67 (6.0)
Other	34 (3.1)
Not stated	227 (20.4)
**Multi-center status, N (%)**	
Yes	370 (33.3)
No	742 (66.7)
**Number of trial locations, median (IQR)**	1 (1, 2)
**Sample Size**^2^, **median (IQR)**	122 (58, 305)
**Study length**^3^, **months, median (IQR)**	24 (12, 38)

^1^ Phase 0 trials included as Phase 1. Trials described as phase I/II categorized as phase II and trials described as phase II/III categorized as phase III.

^2^ Sample size calculations limited to 816 non-cluster RCTs

^3^ Due to missing completion dates, study length available for1092 trials

### Conditions studied in interventional clinical trials

There were 877 trials in our cohort that mapped to one of the 126 WHO disease categories pertaining to elderly persons in high-income countries. These trials studied a total of 54 (43%) of the disease categories (Table 3). The 10 most common disease categories evaluated were: ‘Alzheimer’s disease’ (n = 96 trials), ‘other cardiovascular and circulatory diseases’ (n = 78), ‘falls’ (n = 69), ‘other musculoskeletal disorders’ (n = 62), ‘lower respiratory infections (n = 61), ‘other unintentional injuries’ (n = 42), ‘lung cancer’ (n = 36), ‘diabetes’ (n = 33), ‘leukemia’ (n = 33), and ‘other cancers’ (n = 27) (S1 Table). Fewer than half of the disease categories were also studied in RCTs and drug RCTs while primarily industry-funded trials addressed a quarter of all the conditions. In addition to the conditions that were not studied at all, another 19% (24/126) were studied in five or fewer trials.

**Table 3:** Number of disease categories addressed in trials studying exclusively elderly persons across a total of 126 categories.

Trial type	Disease categories addressed in trials studying exclusively elderly persons, N (%)
**All trials**	54 (42.9) in 877 trials
**RCTs**	49 (38.9) in 641 trials
**Drug RCTs**	41 (32.5) in 233 trials
**Industry-funded RCTs**	32 (25.4) in 142 trials

The burden of disease in the elderly was moderately correlated with the total number of trials (r = 0.53, p<0.001), number of RCTs (r = 0.47, p<0.001), number of drug RCTs (r = 0.40, p = 0.01), and number of industry-funded RCTs (r = 0.47, p = 0.007). There was a weak correlation between burden of disease and number of randomized participants (r = 0.30, p = 0.04).

We found wide variation in the amount of research devoted to different conditions. Among disease categories addressed in at least one trial studying exclusively elderly persons, the number of DALYs per trial ranged from 325 for ‘other nutritional deficiencies’ to 1,677,697 for ‘chronic obstructive pulmonary disease’ (S1 Fig). (‘Other nutritional deficiencies’ represented predominantly malnutrition 15/25 trials] and vitamin D deficiency [9/25 trials]). This represents a 5162-fold range in DALYs per trial. Similarly, when research activity was measured in terms of DALYs per number of randomized participants, there was extreme variation, with the number of DALYs per participant ranging from 1 for ‘varicella’ (which includes varicella zoster) to 14,138 for ‘chronic obstructive pulmonary disease’ (S2 Fig).

There were 5 disease categories for which ≥80% of the disease burden was in persons ≥65 years of age (Table 4). These diseases were addressed in a total of only 117 trials (10.5% of all elderly-only trials) and 87 RCTs (10.4% of all elderly-only RCTs). We found wide variation in the number of trials addressing these conditions with ‘Parkinson’s disease’ not addressed by any trials studying exclusively elderly patients; ‘prostate cancer’, ‘peripheral vascular disease’, and ‘atrial fibrillation’ studied in 11 trials or less; and ‘Alzheimer’s disease and other dementias’ addressed by 96 trials.

**Table 4:** Trials studying disease categories with ≥80% of disease burden among elderly persons.

Disease category	DALYs	% burden of disease in elderly	Number of trials	Number of RCTs	DALYs per trial
Alzheimer’s disease and other dementias	5,253,905	91	96	73	54,728
Parkinson’s disease	744,980	88	0	0	N/A
Prostate cancer	1,521,170	82	5	3	304,234
Atrial fibrillation	1,065,072	81	11	7	96,825
Peripheral vascular disease	291,897	80	5	4	58,379

## Discussion

Only approximately 1% of interventional trials conducted primarily in high-income countries are focused exclusively on elderly persons. Our findings do not support a strong role for these trials in mitigating the problem of insufficient trial evidence on the use of interventions in the elderly. The majority of the trials consisted of small studies conducted at single centers and less than half of all disease categories affecting elderly persons were studied in these focused trials. There appears to be little interest by industry to support such trials, perhaps because this is a high-risk population in terms of adverse events and because there is limited benefit to industry in limiting the age range in which interventions are applied. The amount of research devoted to specific conditions was not strongly correlated with burden of disease in the elderly. Even for the five conditions that affect almost exclusively the elderly, trials limited to the elderly were absent or rare for four of them and more common only for Alzheimer’s disease.

Prior research examining clinical trials in elderly populations has focused on the exclusion of older patients in clinical trials.[4–7,29] Findings have demonstrated that over one third of trials exclude elderly persons by means of arbitrary upper age limits and that this practice is common in trials across a number of disease types, including those directly related to the aging process.[4,5,29,30] Even when there are no age exclusions, many older patients do not meet enrollment criteria due to other factors, resulting in the study of elderly persons that may not resemble the patients encountered in actual clinical practice. [29,30] This has raised concerns about the external validity of clinical trials and the applicability of clinical practice guidelines to elderly patients across a number of domains.[31,32] Trials focusing exclusively on elderly persons might serve to address this issue and increase the clinical evidence available to guide care in the elderly.

Often it is uncertain whether excluding patients from trials based on age is desirable or justified, even when it may serve to increase the enrollment of elderly persons.[29] The current research agenda shows substantial variability across different diseases in this regard. In particular, the two major diseases that affect almost exclusively the elderly (Parkinson’s disease and Alzheimer’s disease) differ substantially in terms of whether investigators choose to conduct trials focused entirely on the elderly. We found no such trials for Parkinson’s disease while there were 96 trials studying exclusively elderly persons for Alzheimer’s disease. This difference may be due to the use of widely used definitions or convenient criteria that define diseases and disease subtypes (e.g. early-onset and late-onset) based on an age cut-off.

A number of factors drive decisions around intervention types, conditions, and populations that are studied in clinical trials. Stakeholders include academic investigators, pharmaceutical companies, and federally funded research groups, all of whom base decisions on a different core of incentives, resources, and access to patients. Patient advocacy groups and public health campaigns may also influence research priorities, contributing to the focus on certain diseases. These stakeholders may need to revisit the need to perform more trials focused on the elderly, especially for diseases that are seen almost exclusively in the elderly.

A few limitations should be considered in interpreting our findings. Our analysis was based on data in ClinicalTrials.gov and relies on accurate and complete reporting by investigators. We were not able to verify or obtain further data and there was some missing information. It is possible that there are trials that enroll exclusively elderly persons even though they do not specify this in their enrollment criteria. To investigate this possibility, we examined a random sample of 100 trials in ClinicalTrials.gov that included participant age information in their trial results section. We did not identify any trials recruiting exclusively elderly persons in this sample, indicating that our trial cohort is likely an accurate description of trials that focus exclusively on the elderly.

Importantly, we should acknowledge that for most, if not for all of the diseases, the number of elderly patients studied in trials that do not include only the elderly is likely to be much larger than the number of elderly persons studied in trials focusing exclusively on the elderly population. Therefore, the conduct of few trials exclusively in the elderly should not be extrapolated to mean necessarily that we have no evidence on how these interventions work in this age group. However, given the very large burden of disease in the elderly and the frequent under-representation of elderly patients in trials that have broad age-related eligibility criteria, the conduct of more elderly-focused trials should be seriously considered by investigators, sponsors, and policy makers seeking to address research needs among elderly persons.

## Supporting Information

S1 Fig DALYs per trial among trials enrolling exclusively elderly persons. Disease labels included for minimum and maximum as well as 10^th^, 25^th^, 50^th^, 75^th^, and 90^th^ percentile of values.
(DOCX)

S2 Fig DALYs per randomized participant among trials enrolling exclusively elderly persons. Disease labels included for minimum and maximum as well as 10^th^, 25^th^, 50^th^, 75^th^, and 90^th^ percentile of values.
(DOCX)

S1 Table Disease categories and trials enrolling exclusively elderly persons, ordered according to DALYs per trial. (DOCX)

## Abbreviations

WHO: World Health Organization
DALY: disability-adjusted life year
RCT: randomized controlled trial
