# Supplementary material for: Prevalence and Characteristics of Interventional Trials Conducted Exclusively in Elderly Persons: A Cross-Sectional Analysis of Registered Clinical Trials
Source: PLoS One. 2016 May 19;11(5):e0155948. doi: 10.1371/journal.pone.0155948 (PMC4873036; doi:10.1371/journal.pone.0155948)
Supplement: S2 Fig — Disease labels included for minimum and maximum as well as 10th, 25th, 50th, 75th, and 90th percentile of values. (DOCX) [file pone.0155948.s002.docx]

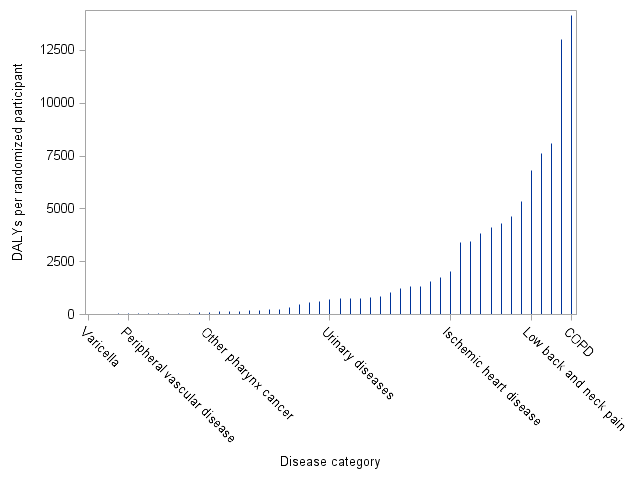


**S2 Fig.** DALYs per randomized participant among trials enrolling exclusively elderly persons. Disease labels included for minimum and maximum as well as 10^th^, 25^th^, 50^th^, 75^th^, and 90^th^ percentile of values.
