# Supplementary material for: Prevalence and Characteristics of Interventional Trials Conducted Exclusively in Elderly Persons: A Cross-Sectional Analysis of Registered Clinical Trials
Source: PLoS One. 2016 May 19;11(5):e0155948. doi: 10.1371/journal.pone.0155948 (PMC4873036; doi:10.1371/journal.pone.0155948)
Supplement: S1 Table — (DOCX) [file pone.0155948.s003.docx]

**S1 Table. Disease categories and trials enrolling exclusively elderly persons, ordered according to DALYs per trial**

| **No.** | **Disease category** | **DALYs** | **Number of**  **trials** | **Number of**  **RCTs** | **Number enrolled in non-cluster RCTs** | **Number of**  **Drug**  **RCTs** | **DALYs**  **per trial** |
| --- | --- | --- | --- | --- | --- | --- | --- |
| 1 | COPD | 5,033,090 | 3 | 3 | 356 | 1 | 1,677,697 |
| 2 | Stroke | 7,856,328 | 6 | 5 | 604 | 2 | 1,309,388 |
| 3 | Ischemic heart disease | 13,538,982 | 11 | 11 | 6,624 | 5 | 1,230,817 |
| 4 | Low back and neck pain | 5,933,368 | 5 | 5 | 873 | 1 | 1,186,674 |
| 5 | Stomach cancer | 1,408,641 | 2 | 2 | 343 | 2 | 704,321 |
| 6 | Kidney cancers | 655,526 | 1 | 0 | 0 | 0 | 655,526 |
| 7 | Hypertensive heart disease | 1,169,079 | 3 | 3 | 1,467 | 1 | 389,693 |
| 8 | Diarrheal diseases | 353,393 | 1 | 1 | 82 | 0 | 353,393 |
| 9 | Prostate cancer | 1,521,170 | 5 | 3 | 284 | 3 | 304,234 |
| 10 | Skin diseases | 1,192,401 | 4 | 3 | 1,529 | 1 | 298,100 |
| 11 | Schizophrenia | 274,830 | 1 | 1 | 36 | 1 | 274,830 |
| 12 | Esophageal cancer | 527,818 | 2 | 0 | 0 | 0 | 263,909 |
| 13 | Sense organ diseases | 2,362,483 | 9 | 4 | 618 | 0 | 262,498 |
| 14 | Chronic kidney disease | 1,995,456 | 8 | 6 | 1,507 | 3 | 249,432 |
| 15 | Ovarian cancer | 426,271 | 2 | 1 | 240 | 1 | 213,136 |
| 16 | Uterine cancer | 205,200 | 1 | 1 | 60 | 0 | 205,200 |
| 17 | Asthma | 565,681 | 3 | 1 | 70 | 0 | 188,560 |
| 18 | Anxiety disorders | 632,278 | 4 | 3 | 841 | 2 | 158,070 |
| 19 | Osteoarthritis | 1,419,763 | 10 | 10 | 1,143 | 3 | 141,976 |
| 20 | Colorectal cancer | 2,687,082 | 19 | 10 | 2,031 | 6 | 141,425 |
| 21 | Oral disorders | 947,113 | 7 | 6 | 275 | 2 | 135,302 |
| 22 | Lung cancer | 4,693,574 | 36 | 18 | 4,383 | 17 | 130,377 |
| 23 | Other respiratory diseases | 371,324 | 3 | 2 | 236 | 0 | 123,775 |
| 24 | Bipolar disorder | 118,825 | 1 | 0 | 0 | 0 | 118,825 |
| 25 | Urinary diseases | 2,160,322 | 19 | 12 | 2,993 | 10 | 113,701 |
| 26 | Diabetes | 3,525,089 | 33 | 30 | 4,635 | 14 | 106,821 |
| 27 | Atrial fibrillation | 1,065,072 | 11 | 7 | 20,051 | 2 | 96,825 |
| 28 | Unipolar depressive disorders | 1,749,468 | 23 | 16 | 4,728 | 9 | 76,064 |
| 29 | Breast cancer | 1,290,127 | 17 | 8 | 2,690 | 5 | 75,890 |
| 30 | Epilepsy | 191,378 | 3 | 1 | 41 | 1 | 63,793 |
| 31 | Peripheral vascular disease | 291,897 | 5 | 4 | 5,294 | 2 | 58,379 |
| 32 | Brain cancer | 438,442 | 8 | 3 | 679 | 3 | 54,805 |
| 33 | Alzheimer's disease and other dementias | 5,253,905 | 96 | 73 | 25,970 | 22 | 54,728 |
| 34 | Adverse medical treatment | 256,186 | 5 | 5 | 2,999 | 0 | 51,237 |
| 35 | Other cancers | 1,373,391 | 27 | 13 | 20,827 | 1 | 50,866 |
| 36 | Lower respiratory infections | 3,069,342 | 61 | 47 | 200,855 | 5 | 50,317 |
| 37 | Other digestive diseases | 547,655 | 11 | 8 | 971 | 4 | 49,787 |
| 38 | Falls | 3,429,966 | 69 | 61 | 14,366 | 5 | 49,710 |
| 39 | Inguinal & femoral hernia | 42,657 | 1 | 0 | 0 | 0 | 42,657 |
| 40 | Other musculoskeletal | 2,468,343 | 62 | 56 | 42,375 | 20 | 39,812 |
| 41 | Myeloma | 389,133 | 10 | 3 | 2,254 | 3 | 38,913 |
| 42 | Other endocrine | 704,738 | 19 | 14 | 4,665 | 7 | 37,091 |
| 43 | Non-Hodgkin lymphoma | 671,133 | 19 | 3 | 790 | 2 | 35,323 |
| 44 | Protein-energy malnutrition | 104,768 | 3 | 3 | 423 | 0 | 34,923 |
| 45 | Other neurological disorders | 592,113 | 21 | 18 | 3,604 | 6 | 28,196 |
| 46 | Other pharynx cancer | 127,355 | 5 | 3 | 1,070 | 1 | 25,471 |
| 47 | Other cardio & circulatory | 1,961,623 | 78 | 58 | 28,891 | 24 | 25,149 |
| 48 | Leukemia | 733,407 | 33 | 14 | 3,962 | 10 | 22,224 |
| 49 | Other infectious diseases | 115,827 | 7 | 7 | 1,967 | 1 | 16,547 |
| 50 | Other unintentional injuries | 556,466 | 42 | 38 | 5,136 | 8 | 13,249 |
| 51 | Encephalitis | 11,070 | 1 | 0 | 0 | 0 | 11,070 |
| 52 | Other mental & behavioral | 132,312 | 14 | 12 | 2,163 | 6 | 9,451 |
| 53 | Varicella | 18,336 | 2 | 2 | 15,262 | 0 | 9,168 |
| 54 | Other nutritional deficiencies | 8,118 | 25 | 23 | 4,636 | 1 | 325 |
| 55 | Pancreatic cancer | 1,291,808 | 0 | 0 | 0 | 0 |  |
| 56 | Liver cancer | 1,095,289 | 0 | 0 | 0 | 0 |  |
| 57 | Road injury | 748,254 | 0 | 0 | 0 | 0 |  |
| 58 | Parkinson's disease | 744,980 | 0 | 0 | 0 | 0 |  |
| 59 | Cardiomyopathy | 713,333 | 0 | 0 | 0 | 0 |  |
| 60 | Rheumatoid arthritis | 683,344 | 0 | 0 | 0 | 0 |  |
| 61 | Bladder cancer | 655,155 | 0 | 0 | 0 | 0 |  |
| 62 | Aortic aneurysm | 610,800 | 0 | 0 | 0 | 0 |  |
| 63 | Self-harm | 533,628 | 0 | 0 | 0 | 0 |  |
| 64 | Interstitial lung diseases | 490,418 | 0 | 0 | 0 | 0 |  |
| 65 | Cirrhosis hepatitis C | 465,356 | 0 | 0 | 0 | 0 |  |
| 66 | Gallbladder cancer | 459,594 | 0 | 0 | 0 | 0 |  |
| 67 | Rheumatic heart disease | 388,481 | 0 | 0 | 0 | 0 |  |
| 68 | Cirrhosis alcohol | 371,736 | 0 | 0 | 0 | 0 |  |
| 69 | Migraine | 326,002 | 0 | 0 | 0 | 0 |  |
| 70 | Pneumoconiosis | 304,585 | 0 | 0 | 0 | 0 |  |
| 71 | Vascular intestinal disorders | 279,128 | 0 | 0 | 0 | 0 |  |
| 72 | Intestinal obstructions | 254,276 | 0 | 0 | 0 | 0 |  |
| 73 | Non-melanoma skin cancer | 234,938 | 0 | 0 | 0 | 0 |  |
| 74 | Alcohol use disorders | 233,788 | 0 | 0 | 0 | 0 |  |
| 75 | Hemoglobinopathies | 223,892 | 0 | 0 | 0 | 0 |  |
| 76 | Gall bladder diseases | 222,629 | 0 | 0 | 0 | 0 |  |
| 77 | Peptic ulcer | 212,471 | 0 | 0 | 0 | 0 |  |
| 78 | Melanoma | 199,955 | 0 | 0 | 0 | 0 |  |
| 79 | Cirrhosis hepatitis B | 199,780 | 0 | 0 | 0 | 0 |  |
| 80 | Mouth cancer | 199,429 | 0 | 0 | 0 | 0 |  |
| 81 | Congenital anomalies | 170,771 | 0 | 0 | 0 | 0 |  |
| 82 | Larynx cancer | 151,822 | 0 | 0 | 0 | 0 |  |
| 83 | Cervical cancer | 149,812 | 0 | 0 | 0 | 0 |  |
| 84 | Tuberculosis | 147,582 | 0 | 0 | 0 | 0 |  |
| 85 | Inflammatory bowel disease | 144,676 | 0 | 0 | 0 | 0 |  |
| 86 | Pancreatitis | 131,932 | 0 | 0 | 0 | 0 |  |
| 87 | Fire | 119,674 | 0 | 0 | 0 | 0 |  |
| 88 | Pervasive developmental disorders | 117,443 | 0 | 0 | 0 | 0 |  |
| 89 | Iron-deficiency anemia | 104,619 | 0 | 0 | 0 | 0 |  |
| 90 | Drowning | 104,026 | 0 | 0 | 0 | 0 |  |
| 91 | Other transport injuries | 85,935 | 0 | 0 | 0 | 0 |  |
| 92 | Meningitis | 84,115 | 0 | 0 | 0 | 0 |  |
| 93 | Otitis media | 82,145 | 0 | 0 | 0 | 0 |  |
| 94 | Endocarditis | 80,143 | 0 | 0 | 0 | 0 |  |
| 95 | Thyroid cancer | 77,337 | 0 | 0 | 0 | 0 |  |
| 96 | Mechanical forces | 73,833 | 0 | 0 | 0 | 0 |  |
| 97 | Cirrhosis other | 73,250 | 0 | 0 | 0 | 0 |  |
| 98 | Multiple sclerosis | 71,481 | 0 | 0 | 0 | 0 |  |
| 99 | Gynecological diseases | 55,227 | 0 | 0 | 0 | 0 |  |
| 100 | Drug use disorders | 53,534 | 0 | 0 | 0 | 0 |  |
| 101 | Tension-type headache | 51,610 | 0 | 0 | 0 | 0 |  |
| 102 | Interpersonal violence | 51,076 | 0 | 0 | 0 | 0 |  |
| 103 | Iodine deficiency | 46,289 | 0 | 0 | 0 | 0 |  |
| 104 | Upper respiratory infections | 38,588 | 0 | 0 | 0 | 0 |  |
| 105 | Poisonings | 38,041 | 0 | 0 | 0 | 0 |  |
| 106 | Eating disorders | 35,945 | 0 | 0 | 0 | 0 |  |
| 107 | Hodgkin's lymphoma | 32,658 | 0 | 0 | 0 | 0 |  |
| 108 | Nasopharynx cancer | 31,456 | 0 | 0 | 0 | 0 |  |
| 109 | HIV/AIDS | 31,271 | 0 | 0 | 0 | 0 |  |
| 110 | Appendicitis | 28,439 | 0 | 0 | 0 | 0 |  |
| 111 | Hepatitis | 27,997 | 0 | 0 | 0 | 0 |  |
| 112 | Gout | 25,372 | 0 | 0 | 0 | 0 |  |
| 113 | Gastrititis & duodenitis | 23,516 | 0 | 0 | 0 | 0 |  |
| 114 | Forces of nature | 22,897 | 0 | 0 | 0 | 0 |  |
| 115 | Animal contact | 11,205 | 0 | 0 | 0 | 0 |  |
| 116 | STDs (excluding HIV/AIDS) | 9,690 | 0 | 0 | 0 | 0 |  |
| 117 | Testicular cancer | 6,353 | 0 | 0 | 0 | 0 |  |
| 118 | Glomerulonephritis | 2,203 | 0 | 0 | 0 | 0 |  |
| 119 | War & legal intervention | 1,100 | 0 | 0 | 0 | 0 |  |
| 120 | Intellectual disability | 657 | 0 | 0 | 0 | 0 |  |
| 121 | Typhoid fevers | 426 | 0 | 0 | 0 | 0 |  |
| 122 | Tetanus | 412 | 0 | 0 | 0 | 0 |  |
| 123 | Diptheria | 0 | 0 | 0 | 0 | 0 |  |
| 124 | Whooping cough | 0 | 0 | 0 | 0 | 0 |  |
| 125 | Measles | 0 | 0 | 0 | 0 | 0 |  |
| 126 | Vitamin A deficiency | 0 | 0 | 0 | 0 | 0 |  |
